# Phylogenetic group distribution and antibiotic resistance of *Escherichia coli* isolates in aquatic environments of a highly populated area

**DOI:** 10.3934/microbiol.2024018

**Published:** 2024-05-09

**Authors:** Rosette Mansour, Mohammad H. El-Dakdouki, Sara Mina

**Affiliations:** 1 Department of Biological Sciences, Faculty of Sciences, Beirut Arab University, P.O. Box 11-5020, Riad El Solh 11072809, Beirut, Lebanon; 2 Department of Chemistry, Faculty of Sciences, Beirut Arab University, P.O. Box 11-5020, Riad El Solh 11072809, Beirut, Lebanon; 3 Department of Medical Laboratory Sciences, Faculty of Health Sciences, Beirut Arab University, P.O. Box 11-5020, Riad El Solh 11072809, Beirut, Lebanon

**Keywords:** Extended-spectrum beta-lactamase, *Escherichia coli*, resistance, water, contamination, phylogroup, Lebanon

## Abstract

**Background:**

Extended-spectrum beta-lactamase (ESBL)-producing *Enterobacteriaceae* including *Escherichia coli* (*E. coli*), are recognized as a global public health threat due to their multidrug-resistant (MDR) phenotypes and their rapid dissemination in aquatic environments. Nevertheless, studies investigating the prevalence and antimicrobial resistance (AMR) profile of ESBL-producing *E. coli* in Lebanese surface water are limited.

**Objective:**

This study aimed to assess the physicochemical properties and microbial contamination load and to determine the distribution of AMR patterns of ESBL-producing *E. coli* in surface water samples from different sites in the North Governorate of Lebanon.

**Methods:**

Water samples were collected from 25 major sites in North Lebanon. These samples were analyzed for the presence of total coliforms, *E. coli*, and fecal enterococci. Phenotypic and genetic characterizations were then performed for *E. coli* isolates to determine their resistance patterns and phylogenetic groups.

**Results:**

Fifty-six samples out of 100 samples were positive for ESBL-producing *E. coli*, mostly harboring bla_CTX-M_ (40/56, 71%) including bla_CTX-M-15_ (33/40, 82%), bla_TEM_ gene (36/56, 64%), bla_SHV_ (20/56, 36%), and bla_OXA_ (16/56, 29%) including bla_OXA-48_ gene (11/16, 69%). Most ESBL-producing *E. coli* isolates belonged to the extra-intestinal pathogenic phylogroup B2 (40/56, 71.4%) while 10/56 (17.9%) belonged to the commensal phylogroup A.

**Conclusion:**

Our results highlight the need to implement effective water monitoring strategies to control transmission of ESBL-producing *E. coli* in surface water and thus reduce the burden on human and animal health.

## 1. Introduction

Bacterial antimicrobial resistance (AMR) has emerged as a global public health concern, especially in clinical and veterinary settings. Recently, increasing attention has been focused on the pervasive distribution of AMR in anthropogenically impacted environments such as air, soil, wastewater, surface water from rivers and lakes, and other recreational waters [1]–[3]. Interestingly, aquatic environments are recognized as one of the natural reservoirs and ideal settings for the acquisition and spread of antibiotic-resistant bacteria (ARB) from urban, industrial, medical, and agricultural waste. Wastewater is a favorable environment for microbial proliferation and gene exchange between species thanks to the plethora of bacteria, high nutrient levels, and optimal temperature [4]. Furthermore, surface waters have been recognized as potential reservoirs for ARB. Studies have shown that an estimated 30–90% of antibiotics administered to humans and animals are excreted as active substances and discharged into the environment [5]. In the aquatic system, these antibiotics may exert selective pressure on bacteria, leading to microbial resistance and serious health consequences [3]. In addition, the potential of disseminating multidrug-resistant bacteria (MDR) into the population is quite significant, posing a serious public health risk resulting from improper sewage disposal and treatment. MDR is defined as acquired non-susceptibility to at least one agent in more than three antimicrobial classes [6]. Interestingly, human behaviors associated with agricultural pollution and urbanization exacerbate water environment pollution and deterioration, worsening river and lake water quality. An estimated 80% of industrial and municipal wastewater worldwide is released into the environment without any previous treatment, which harms ecosystems and poses risks to human and animal health. Because of the severe lack of sanitation and wastewater treatment systems, this percentage is greater in developing countries [7].

*Enterobacteriaceae*, a large family of Gram-negative and non-spore-forming rods, includes potential pathogens of several genera such as *Escherichia*, *Enterobacter*, *Klebsiella*, *Proteus*, *Citrobacter*, *Serratia*, *Salmonella*, and *Shigella*. These bacteria can be transmitted through fecal material and can be found in various environments such as soil, water, and food [8]. *Escherichia coli* (*E. coli*), part of the normal flora of the gastrointestinal tract, spreads in natural environments either directly through fecal waste or indirectly through poorly managed wastewater, making it a primary indicator of fecal bacterial contamination in water, food, and soil [4]. It usually contaminates water systems by various routes including human activities and animal, hospital, and municipal discharges. An increased prevalence of extended-spectrum β-lactamases (ESBLs) has been recently reported [9]. ESBLs are a cluster of enzymes that effectively break down third-generation oxyimino-cephalosporins such as cefotaxime, ceftazidime, and ceftriaxone, as well as monobactams like aztreonam, but not cephamycins (cefoxitin, cefotetan) and carbapenems (imipenem, ertapenem, biapenem, meropenem, and doripenem) [10]. Most ESBLs are encoded by antibiotic resistance genes (ARGs) frequently found on mobile genetic elements of a variety of Gram-negative bacteria. Biofilms of the aquatic system can serve as hotspots for disseminating these ARGs, contributing to increased pathogenicity and antimicrobial resistance [11]. More than 400 ESBL subtypes have been identified so far, most commonly derived from the TEM (Temoniera), SHV (Sulfhydryl Variable), OXA (Oxacillinase), and CTX-M (Cefotaximase-München) types [12]. Notably, the CTX-M type (mainly CTX-M-15) is among the most prevalent of ESBL genes described in communities, clinical settings, and water networks, followed by the TEM and SHV types [13]. ESBL-producing *E. coli* has been found in recreational waters, wastewater, and surface water from rivers and lakes over the past decade [14]. Furthermore, the discovery of ESBL-producing *E. coli* in companions, livestock, and wild animals has hypothesized its role in spreading ESBL-producing bacteria in the environment [15].

Lebanon, a developing country on the Mediterranean Sea, has struggled with post-civil war infrastructure, waste, and water management issues, in addition to a population growth since 2011 including an influx of 1.5 million refugees that exerted a significant strain on existing resources and services [16]. Surface water in Lebanon is intended for human consumption, recreational activities, electricity generation, and irrigation [17]. In 2016, only 58.54% of buildings were properly connected to a sewerage system; the remaining buildings either used septic tanks or cesspools or directly discharged untreated sewage into the aquatic system. It was also reported that 92% of the collected wastewater is discarded into aquatic environments without any prior treatment [18]. The majority of water sources are contaminated with industrial waste and sewage, posing a significant risk to public health and the economy. Many studies conducted in Lebanon showed a deterioration pattern in domestic water and seawater quality [19],[20]. In addition, recent reports have highlighted the contamination of spring and well waters intended for human consumption, as well as estuaries that harbor unique plant and animal communities [21]. ESBL-producing *E. coli* has been also isolated from river effluents in Lebanon, including refugee camps, highlighting the magnitude of the problem associated with the dissemination of drug-resistant Gram-negative bacteria [16]. Thus, the present study aimed to assess the water quality and occurrence of antibiotic-resistant bacteria in water samples collected from different surface water sites in the North Governorate of Lebanon and to estimate the occurrence and molecular characterization of ESBL-producing *E. coli* isolates.

## 2. Materials and methods

### 2.1. Sample collection

**Figure 1.**
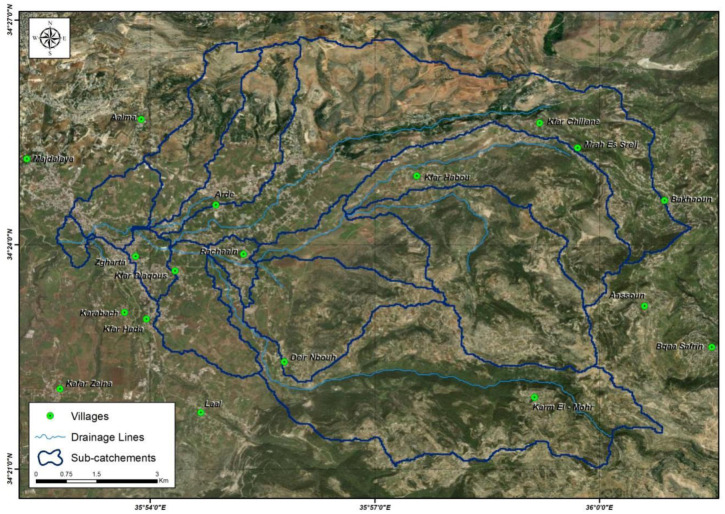
Map of the study area in North Lebanon.

A total of 100 water samples were collected from 25 different surface water sites (spaced approximately 200 m apart) in the North Governorate of Lebanon between July 2020 and April 2021. The sampling sites were chosen to achieve a possible compromise between the representativeness of the contaminated areas and operational feasibility. The selected sites are presented in Figure 1 and designated as follows: upstream area (S1 to S5), midstream area (S6 to S14), and downstream area (S15 to S25). Many sites (Al Merdechyeh, Rachaaine, Kfar Dlaqous, Zgharta, and Tripoli) were in the discontinuous urbanized upstream area; this area is influenced by agricultural and anthropogenic activities, in addition to high population size estimated at 331,500 [22]. A 50 mL sample was collected from each site in a sterile glass bottle, kept at 4 °C, and processed within six hours of sampling.

### 2.2. Physiochemical characterization of water samples

Physicochemical parameters including temperature and pH were measured. Turbidity in surface water indicates the presence of suspended solids and organic matter. It was measured by a Lovibond water testing turbidimeter. Dissolved oxygen (DO) is an important indicator of water quality by measuring oxygen concentration. A Thermo Orion was employed to evaluate the DO and biochemical oxygen demand (BOD) for 5 days.

### 2.3. Microbiological analysis, bacterial isolation, and identification

Samples were processed to determine the concentrations of total coliforms (TC), fecal coliforms (FC), and enterococci (ENT), using a membrane filtration method for testing TC (protocol 9222B), FC (protocol 9222D17), and ENT (US EPA Method 1600) [23]. Membrane filters were then transferred to Petri dishes and placed on Endo agar, membrane fecal coliform agar, and Enterococcus selective agar (Sigma-Aldrich, USA) for TC, FC, and ENT bacteria respectively. They were incubated for 24 h at 37 °C for the total coliforms. Fecal coliforms were incubated at 44.5 °C for 24 h. Each isolated colony was examined for its morphological characteristics, and the viable bacterial count was enumerated in terms of colony-forming units (CFU) per 100 milliliters. The French SEQ-EAU-2003 and the US EPA standards [24] were used to evaluate irrigation and recreational water quality, respectively. According to SEQ-EAU-2003, the acceptable limit for thermo-tolerant fecal coliforms (fecal coliforms or *E. coli*) is 100 CFU/100 mL, which aligns with the standards set by other European Union countries [25]. Thus, we adopted these standards to assess the suitability of water sources for irrigation and recreation in Lebanon.

*Enterobacteriaceae* resistant to broad-spectrum cephalosporins were selected by directly plating 100 µL of water on ESBL ChromoSelect agar plates (Sigma-Aldrich, USA) and incubated at 37 °C for 24 h. Typical *E. coli* colonies were randomly picked from selective plates and identified using Api 20E (BioMerieux SA, Lyon, France).

### 2.4. Antibiotic susceptibility testing

Antibiotic susceptibility was tested using 27 different antibiotics belonging to 11 different antibiotic classes, including the penicillins amoxicillin (AMX, 25 µg), ticarcillin (TIC, 75 µg), and piperacillin (PIP, 100 µg); the beta-lactamase inhibitor combinations amoxicillin/clavulanic acid (AMC, 20 µg/10 µg), ticarcillin/clavulanic acid (TIM, 75 µg/10 µg ), and piperacillin–tazobactam (TZP, 100 µg/10 µg); the cephalosporins cefuroxime (CXM, 30 µg), cefoxitin (FOX, 30 µg), cefotaxime (CTX, 30 µg), ceftriaxone (CRO, 30 µg), ceftazidime (CAZ, 30 µg), cefepime (FEP, 30 µg), and cefixime (CFM, 5 µg); the carbapenems imipenem (IPM, 10 µg) and ertapenem (ETP, 10 µg); the monobactams aztreonam (ATM, 30 µg); the aminoglycosides streptomycin (SMN, 10 µg), tobramycin (TOB, 30 µg), gentamicin (GEN, 10 µg), and amikacin (AMK, 30 µg); the tetracycline tetracycline (TET, 30 µg); the fluoroquinolone ciprofloxacin (CIP, 5 µg); the sulfonamides sulfonamide (SSS, 300 µg) and trimethoprim/sulfamethoxazole (SXT, 25 µg); the phenicol chloramphenicol (CHL, 30 µg); the quinolones nalidixic acid (NAL, 30 µg); and the non-sulfonamide trimethoprim (TMP, 5 µg) (Bio-Rad, USA). ESBL production, when suspected, was confirmed by the double-disc synergy test, with third-generation cephalosporins (cefotaxime, ceftazidime, and cefixime). Results were interpreted according to the Clinical Laboratory Standards Institute guidelines [26]. Bacteria were classified as susceptible, intermediate, or resistant according to the approved clinical breakpoints. *E. coli* ATCC 25922 was used for quality control.

Antibiotic resistance profiles of ESBL-producing *E. coli* isolates were performed by the disc diffusion method according to the guidelines of the Antibiogram Committee of the French Society for Microbiology (https://www.sfm-microbiologie.org/).

### 2.5. Phylogenetic typing and molecular characterization of ESBL-producing isolates

Genomic DNA extraction was performed using a NucleoSpin Tissue kit (Macherey-Nagel, Hoerdt, France) for each isolate. The genomic DNA samples were stored at −20 °C for downstream molecular analysis.

Phylogenetic typing was performed to determine the main phylogenetic groups (A, B1, B2, C, D, E, and F) of ESBL-producing *E. coli* isolates by polymerase chain reaction (PCR) amplification of *chuA*, *yjaA*, *tspE4C2*, and arpA genes as described previously [27]. The presence of β-lactamase-encoding genes was tested in all ESBL-producing *E. coli* isolates by PCR amplification of bla_TEM_, bla_SHV_, bla_CTX-M_, bla_CTX-M15_, bla_OXA_, and bla_OXA-48_ genes (Table 1) [16],[28],[29]. The PCR mixture for each reaction contained 12.5 µL of 2 × REDTaq® ReadyMix™ (Sigma-Aldrich, USA), 1 µL of each primer (10 µM), 1 µL of template DNA, and double-distilled water (ddH_2_O) to a final volume of 25 µL. The amplification products were separated in 2% agarose gel containing ethidium bromide and then visualized under ultraviolet (UV) light.

**Table 1.** List of target genes and primer sequences used for phylogenetic group typing and antibiotic resistance detection in ESBL-producing *E. coli*.

PCR target		Primer sequence (5′-3′)		Annealing Temperature (°C)	Length (bp)	Reference
Phylogenetic	ChuA	chuA.1b	ATGGTACCGGACGAACCAAC	59	288	[27]
		chuA.2	TGCCGCCAGTACCAAAGACA		
	yjaA	yjaA.1b	CAAACGTGAAGTGTCAGGAG	59	211
		yjaA.2b	AATGCGTTCCTCAACCTGTG		
	TspE4.C	TspE4C2.1	CACTATTCGTAAGGTCATCC	59	152
		TspE4C2.2b	AGTTTATCGCTGCGGGTCGC		
	arpA	AceK.f	AACGCTATTCGCCAGCTTGC	59	400
		ArpA1.r	TCTCCCCATACCGTACGCTA		

β-lactam resistance	bla_TEM_	TemA1	ATAAAATTCTTGAAGAC	42	1075	[16]
		TemB1	TTACCAATGCTTAATCA		
	bla_SHV_	SHV-F	CACTCAAGGATGTATTGTG	49	822
		SHV-R	TTAGCGTTGCCAGTGCTCG		
	bla_CTX-M_	CTX-C1	ATGTGCAGCACCAGTAAAGT	54	545
		CTX-C2	ACCGCGATATCGTTGGTGG		
	bla_OXA_	OXA-F	ACACAATACATATCAACTTCGC	61	813
		OXA-R	AGTGTGTTTAGAATGGTGATC		
	bla_CTX-M-15_	F	CACACGTGGAATTTAGGGACT	50	995	[28]
		R	GCCGTCTAAGGCGATAAACA		

Carbapenem resistance	bla_OXA-48_	F	GCTTGATCGCCCTCGATT	60	238	[29]
		R	GATTTGCTCCGTGGCCGAAA		

### 2.6. Statistical analysis

Data entry and descriptive statistics were performed using IBM SPSS (Statistical Package for the Social Sciences) version 29. Student's t-test was performed for means comparison. A p-value ≤ 0.05 was considered significant.

## 3. Results

### 3.1. Physicochemical characterization and bacteriological analysis of water samples

Physicochemical parameters were measured in the water samples. The mean values of these parameters are presented in Table 2. Recorded temperatures ranged from 17.2 to 20.8 °C during the dry season and from 11.8 to 17.2 °C during the wet season. The recorded pH levels of all sites were within the permissible limits (6.5 to 8.5) set by the World Health Organization (WHO) for potable water [30]. The upstream water samples (S1 to S10) had the lowest turbidity, while the downstream samples (S15 to S25) exceeded the WHO guideline value of 5 NTU. Moreover, the highest average of DO levels (10.64) was observed during the dry sample period. However, the BOD5 content was lower during the dry season (2.76) than in the wet season (3.1).

The results of microbial analysis at the different sampling sites showed that total coliforms ranged from 1.9 × 10^1^ ± 1.7 × 10^1^ to 5.3 × 10^6^ ± 7.8 × 10^6^ CFU/100 mL. In contrast, fecal coliform levels ranged from 5.4 ± 0.58 to 1.6 × 10^6^ ± 2.1 × 10^6^ CFU/100 mL during both seasons. *E. coli* concentrations ranged from 5.0 ± 4.2 to 3.4 × 10^5^ ± 4.6 × 10^5^ CFU/100 mL, and the counts of fecal enterococci ranged from 0 to 5.1 × 10^4^ ± 5.9 × 10^4^ CFU/100 mL. A significant difference in bacterial load was recorded among the different sampling sites (p < 0.001).

Fecal coliforms were detected in 98/100 (98%) of water samples and 84/100 (84%) of sampling sites. In positive sites, the average number of fecal coliforms ranged from 1 × 10^0^ to 4.7 × 10^6^ CFU/100 mL. *E. coli* isolates were recovered from 90/100 (90%) of water samples. *E. coli* concentration ranged from 1 to 1 × 10^6^ CFU/100 mL in the positive sites. The highest average counts of fecal coliforms (1.6 × 10^6^ ± 2.1 × 10^6^ CFU/100 mL) and *E. coli* (3.4 × 10^5^ ± 4.6 × 10^5^) were recorded in samples collected from the midstream and downstream sites (S6 to S25). *E. coli* and fecal coliform counts were then compared to the SEQ-EAU standard (100 CFU/100 mL) for irrigation water quality. Results showed that water samples exceeded the standard based on the standard related to fecal coliforms (84/100, 84%) and *E. coli* counts (80/100, 80%). Overall, the majority of these samples, collected from the midstream and downstream areas, were present in regions with relatively concentrated agricultural practices; only upstream samples were found to be acceptable.

The suitability of water for recreational activities was assessed through the US EPA standard based on a threshold of fecal coliforms of 800 CFU/100 mL. Subsequently, 70/100 (70%) of the samples and 80/100 (80%) of the sites exceeded the recommended safety standard for recreational usage. Additionally, it was noted that samples collected from midstream and downstream sites exceeded the standard (S6 to S25).

**Table 2.** Results showing mean ± standard deviation of microbial and chemical indicator counts from the sampling sites.

	Total Coliforms CFU/100 mL	Fecal Coliforms CFU/100 mL	Enterococci CFU/100 mL	*E. coli* CFU/100 mL	pH	DO mg/L	BOD 5 mg/L	Temperature (°C)	Turbidity NTU
S1	1.9 × 10^1^ ± 1.7 × 10^1^	5.4 ± 0.58	0 ± 0	5.0 ± 4.2	7.9 ± 0.1	10.9 ± 0.5	0.8 ± 0.4	14.5 ± 5.8	0.06 ± 0.01
S2	1.6 × 10^2^ ± 2.4 × 10^2^	10.25 ± 9.74	1.25 ± 1.9	1.4 ± 1.7 × 10^1^	7.8 ± 0.3	10.9 ± 0.3	1.5 ± 0.9	14.9 ± 4.5	0.56 ± 0.24
S3	3.5 × 10^1^ ± 3.3 × 10^1^	9.8 ± 6.9	4.3 ± 4.3	1.6 × 10^1^ ± 1.8 × 10^1^	8 ± 0.3	10.4 ± 1.7	1.8 ± 1.5	17.9 ± 2.7	0.6 ± 0.3
S4	3.6 × 10^1^ ± 3.9 × 10^1^	7.5 ± 6	3.8 ± 4.3	8 ± 8.5	7.9 ± 0.3	10.6 ± 2	1.9 ± 1.5	16.7 ± 3.3	0.56 ± 0.23
S5	1.4 × 10^3^ ± 1.2 × 10^3^	82 ± 36.2	4.5 × 10^1^ ± 3.6 × 10^1^	5 × 10^2^ ± 4.5 × 10^2^	7.9 ± 0.4	10.6 ± 2.5	1.3 ± 1	14.9 ± 4.6	1.05 ± 0.57
S6	9.9 × 10^3^ ± 6.0 × 10^3^	4.1 × 10^3^ ± 4.1 × 10^3^	1.2 × 10^2^ ± 7.5 × 10^1^	4 × 10^3^ ± 3.7 × 10^3^	7.6 ± 0.3	10.2 ± 1.3	1 ± 0.4	15.9 ± 4.1	0.94 ± 0.37
S7	5.7 × 10^3^ ± 5.0 × 10^3^	4.6 × 10^3^ ± 2.5 × 10^3^	3.2 × 10^2^ ± 3.4 × 10^2^	4 × 10^3^ ± 4.1 × 10^3^	7.7 ± 0.2	10.7 ± 1.5	1 ± 0.4	17.6 ± 2.1	1.43 ± 0.59
S8	1.0 × 10^4^ ± 5.9 × 10^3^	7.9 × 10^3^ ± 1.7 × 10^3^	2.2 × 10^2^ ± 2 × 10^2^	5.4 × 10^3^ ± 3 × 10^3^	7.7 ± 0.3	10.5 ± 0.9	1 ± 0.6	17.5 ± 3	1.5 ± 0.5
S9	2.1 × 10^4^ ± 8.7 × 10^3^	4.6 × 10^3^ ± 4.2 × 10^3^	4.3 × 10^2^ ± 5.2 × 10^2^	1.1 × 10^3^ ± 6.8 × 10^3^	7.8 ± 0.3	10.5 ± 0.5	1.1 ± 0.8	17.9 ± 3	1.5 ± 0.54
S10	1.6 × 10^4^ ± 1.3 × 10^4^	5.7 × 10^3^ ± 5.4 × 10^3^	2 × 10^2^ ± 1.5 × 10^2^	3.4 × 10^3^ ± 2.2 × 10^3^	7.8 ± 0.1	10.9 ± 1.3	1.9 ± 1.1	17.5 ± 3.8	1.57 ± 0.42
S11	4.3 × 10^4^ ± 3.9 × 10^4^	2.4 × 10^4^ ± 2 × 10^3^	1.3 × 10^3^ ± 1.5 × 10^3^	2.4 × 10^4^ ± 2.4 × 10^4^	7.9 ± 0.1	10.6 ± 1.5	1.5 ± 0.6	17.8 ± 3.9	1.74 ± 0.12
S12	1.0 × 10^5^ ± 9.8 × 10^4^	3.4 × 10^4^ ± 4.7 × 10^4^	6.1 × 10 ± 5.5 × 10^3^	5.8 × 10^4^ ± 5.9 × 10^4^	7.9 ± 0.2	10.4 ± 0.8	1.7 ± 0.3	16.3 ± 4.1	2.36 ± 0.5
S13	2.0 × 10^5^ ± 2.1 × 10^5^	4.5 × 10^4^ ± 4.3 × 10^4^	6 × 10^3^ ± 4.9 × 10^3^	1.8 × 10^4^ ± 1.5 × 10^4^	7.9 ± 0.2	10.4 ± 1.2	1.2 ± 0.3	17 ± 3.9	2.36 ± 0.6
S14	3.0 × 10^5^ ± 3.5 × 10^5^	5.4 × 10^4^ ± 5.3 × 10^4^	5.9 × 10^3^ ± 4.9 × 10^3^	2 × 10^4^ ± 2.5 × 10^4^	8 ± 0.5	10.4 ± 2	2.5 ± 1.3	17.8 ± 4.4	2.39 ± 0.7
S15	1.0 × 10^6^ ± 6.3 × 10^5^	1.4 × 10^5^ ± 5.1 × 10^4^	2.6 × 10^4^ ± 2.1 × 10^4^	3.8 × 10^4^ ± 2 × 10^4^	7.7 ± 0.2	9.1 ± 3.2	4.4 ± 1.5	19.4 ± 3.4	3.69 ± 1.65
S16	1.5 × 10^5^ ± 1.1 × 10^5^	2.1 × 10^4^ ± 3.3 × 10^4^	2.9 × 10^4^ ± 2.7 × 10^4^	1.1 × 10^4^ ± 8.7 × 10^4^	7.7 ± 0.2	9.6 ± 0.6	3.8 ± 1.8	17.8 ± 2.3	7.69 ± 5.15
S17	8.9 × 10^5^ ± 9.1 × 10^5^	9.3 × 10^4^ ± 8.7 × 10^4^	4.5 × 10^4^ ± 4.5 × 10^4^	3 × 10^4^ ± 2.5 × 10^4^	7.8 ± 0.1	8.1 ± 1.6	4.3 ± 0.8	17.6 ± 3.7	6.15 ± 3.25
S18	2.4 × 10^6^ ± 1.7 × 10^6^	3.6 × 10^5^ ± 3.7 × 10^5^	7.8 × 10^4^ ± 6.8 × 10^4^	1.2 × 10^5^ ± 7.8 × 10^5^	7.9 ± 0.4	7.1 ± 3.3	5.7 ± 1.7	17.5 ± 4.5	4.94 ± 1.97
S19	3.4 × 10^6^ ± 3.2 × 10^6^	1.2 × 10^6^ ± 1.4 × 10^6^	9.3 × 10^4^ ± 8 × 10^4^	2.4 × 10^5^ ± 2.6 × 10^5^	7.9 ± 0.4	7.8 ± 2.2	4.5 ± 1.4	16.4 ± 3.7	5.07 ± 2.2
S20	5.3 × 10^6^ ± 7.8 × 10^6^	1.6 × 10^6^ ± 2.1 × 10^6^	1.3 × 10^5^ ± 1.2 × 10^5^	3.4 × 10^5^ ± 4.6 × 10^5^	7.9 ± 0.4	8.6 ± 1.6	5.8 ± 1.2	17.9 ± 3.4	5.07 ± 2.92
S21	3.3 × 10^6^ ± 5.5 × 10^6^	2.1 × 10^5^ ± 2.1 × 10^5^	5.1 × 10^4^ ± 3.4 × 10^4^	2.9 × 10^5^ ± 4.9 × 10^5^	7.6 ± 0.3	8.7 ± 3.6	5.1 ± 1.1	16.4 ± 3.7	5.29 ± 2.53
S22	1.7 × 10^6^ ± 1.9 × 10^6^	2.1 × 10^5^ ± 1.1 × 10^5^	4.2 × 10^4^ ± 2.7 × 10^4^	1.5 × 10^5^ ± 2.1 × 10^5^	7.7 ± 0.1	8.5 ± 2	4.3 ± 1.3	17.6 ± 3.8	6.74 ± 2.26
S23	1.3 × 10^6^ ± 8.4 × 10^5^	2.5 × 10^5^ ± 1 × 10^5^	3.5 × 10^4^ ± 1.1 × 10^4^	1.7 × 10^5^ ± 1.8 × 10^5^	7.8 ± 0.3	8.4 ± 1	5.5 ± 1	18.9 ± 4.2	9.17 ± 3.89
S24	1.3 × 10^6^ ± 4.5 × 10^5^	3.3 × 10^5^ ± 2.7 × 10^5^	3.9 × 10^4^ ± 3.3 × 10^4^	6 × 10^4^ ± 2.4 × 10^4^	7.7 ± 0.1	8.8 ± 0.6	4.5 ± 1.1	18.1 ± 4.1	11.36 ± 2.81
S25	1.1 × 10^6^ ± 5.5 × 10^5^	3.7 × 10^5^ ± 4.2 × 10^5^	5.1 × 10^4^ ± 5.9 × 10^4^	6.2 × 10^4^ ± 3.7 × 10^4^	7.6 ± 0.2	9.5 ± 0.9	4.8 ± 1.8	18.7 ± 4.6	18.86 ± 11.01

### 3.2. Correlations between various water quality parameters during the dry and wet season

A consistent pattern of strong positive correlations between bacterial contamination indicators (total coliforms, fecal coliforms, *E. coli*, and Enterococci) was observed in both seasons. DO consistently showed negative correlations with bacterial contamination indicators. BOD5 showed positive correlations with bacterial indicators, turbidity, and temperature.

Table 3 summarizes the correlation coefficients between the various water quality parameters during the dry and wet seasons.

During the dry season, total coliform and *E. coli* showed a strong positive correlation with BOD5 (r = 0.766, r = 0.760 respectively) and positive moderate correlation with turbidity (r = 0.497, r = 0.415). Fecal coliforms showed a very strong positive correlation with Enterococci (r = 0.878) and BOD5 (r = 0.669), and a moderate correlation with turbidity (r = 0.397). Negative correlations were observed between DO and total coliforms (r = −0.646), *E. coli* (r = −0.596), and fecal coliforms (r = −0.569). BOD5 showed a positive correlation with temperature (r = 0.537) and a negative correlation with pH (r = −0.487). A strong positive correlation was observed between pH and *E. coli* (r = 0.704), but not with fecal coliforms, fecal enterococci, and total coliforms. During the wet season, total coliforms showed a strong positive correlation with BOD5 (r = 0.719) and a weak positive correlation with turbidity (r = 0.288). A strong positive correlation was observed between *E. coli* and BOD5 (r = 0.736) and a moderate correlation between *E. coli* and turbidity (r = 0.478). Fecal coliform showed a moderate positive correlation with BOD5 (r = 0.539) and a weak correlation with turbidity (r = 0.256). Enterococci showed a very strong positive correlation with BOD5 (r = 0.875) and a moderate correlation with turbidity (r = 0.549). DO had negative correlations with total coliforms (r = −0.674), *E. coli* (r = −0.705), fecal coliforms (r = −0.444), and Enterococci (r = −0.800).

### 3.3. Antibiotic resistance profiles of ESBL-producing E. coli isolates

**Figure 2.**
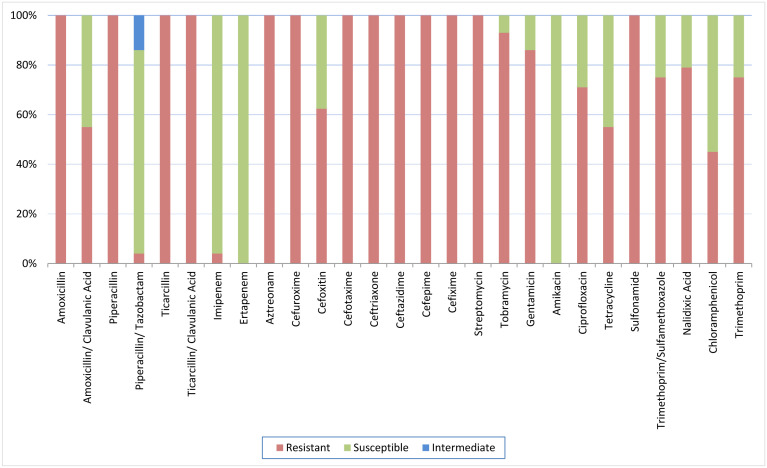
Percentages of resistance against 27 tested antibiotics among ESBL-producing *E. coli* (n = 56) in water samples.

Of the 100 water samples, 56 ESBL-producing *E. coli* isolates were recovered. Resistance to penicillins (amoxicillin, ticarcillin, and piperacillin) was detected in all ESBL-producing *E. coli* isolates. Resistance to beta-lactamase inhibitor combinations was also observed including amoxicillin/clavulanic acid (31/56, 55%), ticarcillin/clavulanic acid (56/56, 100%), and piperacillin–tazobactam (46/56, 82%). All the ESBL-producing *E. coli* isolates were resistant to at least six out of the seven tested cephalosporins, with 35/56 (63%) exhibiting additional resistance to cefoxitin. All the ESBL-producing *E. coli* isolates were also resistant to monobactam, aztreonam, amikacin, ertapenem, and sulfonamide. Different levels of resistance were observed for the remaining antibiotics, including aminoglycosides (streptomycin (56/56, 100%), tobramycin (52/56, 93%), gentamicin (48/56, 86%)), fluoroquinolones (ciprofloxacin 40/56, 71%), and trimethoprim/sulfamethoxazole (42/56, 75%). Furthermore, only 2/56 (4%) of the ESBL-producing *E. coli* isolates were found to be resistant to imipenem. Additional resistance patterns are shown in Figure 2.

**Table 3.** Pearson correlation matrix of various water quality parameters during the dry and wet seasons.

		Total Coliforms	Fecal Coliforms	Enterococci	*E. coli*	pH	DO	BOD 5	Temperature	Turbidity
Total coliforms	dry season	1	0.940**	0.757**	0.895**	−0.368	−0.646**	0.766**	0.363	0.497*
	wet season	1	0.862**	0.786**	0.807**	0.142	−0.674**	0.719**	0.144	0.288
Fecal coliforms	dry season	0.940**	1	0.878**	0.844**	−0.394	−0.569**	0.669**	0.234	0.397*
	wet season	0.862**	1	0.755**	0.813**	0.289	−0.444*	0.539**	0.141	0.256
Enterococci	dry season	0.757**	0.878**	1	0.729**	−0.511**	−0.566**	0.717**	0.199	0.445*
	wet season	0.786**	0.755**	1	0.773**	0.178	−0.800**	0.875**	0.210	0.549**
*E. coli*	dry season	0.895**	0.844**	0.729**	1	0.704**	−0.596**	0.760**	0.363	0.415*
	wet season	0.807**	0.813**	0.773**	1	0.715**	−0.705**	0.736**	0.117	0.478*
pH	dry season	−0.368	−0.394	−0.511**	0.704**	1	0.598**	−0.487*	−0.178	−0.432*
	wet season	0.142	0.289	0.178	0.715**	1	−0.158	−0.037	−0.175	−0.444*
DO	dry season	−0.646**	−0.569**	−0.566**	−0.596**	0.598**	1	−0.789**	−0.459*	−0.732**
	wet season	−0.674**	−0.444*	−0.800**	−0.705**	−0.158	1	−0.783**	−0.217	−0.315
BOD 5	dry season	0.766**	0.669**	0.717**	0.760**	−0.487*	−0.789**	1	0.537**	0.648**
	wet season	0.719**	0.539**	0.875**	0.736**	−0.037	−0.783**	1	0.415*	0.674**
Tem	dry season	0.363	0.234	0.199	0.363	−0.178	−0.459*	0.537**	1	0.671**
	wet season	0.144	0.141	0.210	0.117	−0.175	−0.217	0.415*	1	0.378
Turbidity	dry season	0.497*	0.397*	0.445*	0.415*	−0.432*	−0.732**	0.648**	0.671**	1
	wet season	0.288	0.256	0.549**	0.478*	−0.444*	−0.315	0.674**	0.378	1

* significant Pearson correlation

### 3.4. Phylogenetic typing and ESBL resistance genes

The predominant phylogenetic group in the 56 ESBL-producing *E*. *coli* isolates was B2 (40/56, 71.4%), followed by A (10/56, 17.9%), non-typeable (NT) (3/56, 5.4%), D (2/56, 3.6%), and E (1/56, 1.8%). Among the B2 phylogroup isolates (n = 40), 25/40 (62.5%) possessed bla_CTX-M_, 14/50 (35%) possessed bla_TEM_, 26/40 (65%) possessed bla_SHV_, and 11/40 (27.5%) possessed the bla_OXA_ gene. Out of the A phylogroup isolates (n = 10), 8/10 (80%) harbored bla_CTX-M_, 7/10 (70%) harbored bla_TEM_, 3/10 (30%) harbored bla_SHV_, and 3/10 (30%) harbored the bla_OXA_ gene (Table 4).

ESBL genes were detected in all 56 ESBL-producing *E*. *coli* isolates. The bla_CTX-M_ gene (40/56, 71%) was the most prevalent ARG, followed by bla_TEM_ (36/56, 64%), bla_SHV_ (20/56, 36%), and bla_OXA_ (16/56, 29%). Co-occurrence of bla_CTX-M_ with other ESBL genes (bla_TEM_, bla_SHV_, or bla_OXA_) was also detected in 31/56 (55%) of the MDR isolates in this study. Among them, bla_CTX-M_ was co-carried with bla_TEM_ (13/56, 23%), bla_SHV_ (6/56, 11%), and bla_OXA_ (2/56, 4%) ARGs. In addition, 5/56 (9%) of the MDR isolates carried the 4 genes, 7/56 (12%) carried bla_CTX-M_ alone, 5/56 (9%) carried bla_TEM_ alone, and none of the isolates carried bla_SHV_ or bla_OXA_ alone (Table 4).

**Table 4.**
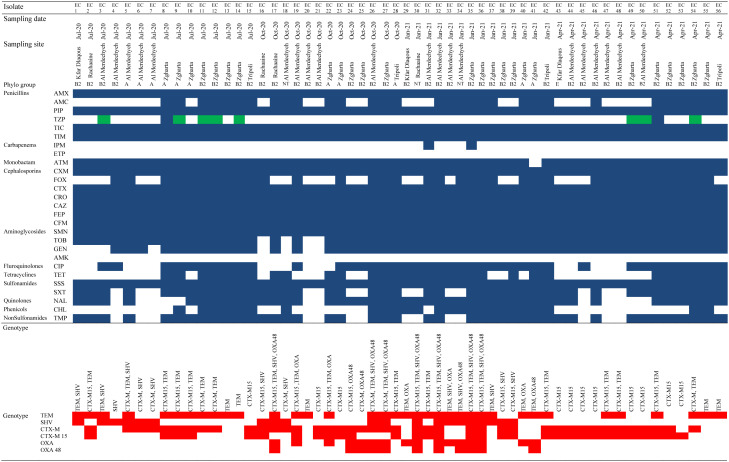
Distribution of antibiotic resistance genes among ESBL-producing *E. coli* isolated from water samples and correlation with phylogenetic groups and resistance phenotypes.

AMX = Amoxicillin, AMC = Amoxicillin/Clavulanic Acid, PIP = Piperacillin, TZP = Piperacillin/Tazobactam, TIC = Ticarcillin, TIM = Ticarcillin/Clavulanic Acid, IPM = Imipenem, ETP = Ertapenem, ATM = Aztreonam, CXM = Cefuroxime, FOX = Cefoxitin, CTX = Cefotaxime, CRO = Ceftriaxone, CAZ = Ceftazidime, FEP = Cefepime, CFM = Cefixime, SMN = Streptomycin, TOB = Tobramycin, GEN = Gentamicin, AMK = Amikacin, CIP = Ciprofloxacin, TET = Tetracycline, SSS = Sulfonamide, SXT = Trimethoprim/Sulfamethoxazole, NAL = Nalidixic Acid, CHL = Chloramphenicol, TMP = Trimethoprim. NT = Non-Typeable. Dark blue = resistant, white = susceptible, green = intermediate, and red = present.

## 4. Discussion

It is widely acknowledged that untreated sewage and various agricultural and industrial pollutants have severe impacts on aquatic environments. However, there is a scarcity of studies focusing on fecal pollution and microbial safety of surface water in Lebanon, and in the Northern region specifically. Therefore, monitoring the quality of water used for irrigation is of utmost importance to control the spread of diseases in Lebanon, which is particularly vulnerable due to ongoing medical and economic crises.

The present study revealed that 98/100 (98%) of the water samples contained fecal coliforms including *E. coli* (90/100, 90%). When considering both fecal bacteria, it was observed that 80/100 (80%) and 84/100 (84%) of the water samples exceeded the SEQ-EAU-2003 standard for irrigation and recreational activities. The widespread presence of fecal indicators is not surprising, considering that 92% of the collected wastewater in Lebanon is discharged untreated into aquatic environments [18]. Our findings are higher than those reported in a previous study conducted across all rivers in Lebanon by Dagher et al. [31]. Pollution levels, expressed by the density of fecal indicators, tend to increase in densely populated and agriculturally/industrially active sites. This can be attributed to the fact that surface water carries sewage from different villages, as well as effluents from informal refugee settlements, various industries, and poultry farms. Notably, surface water serves as the primary source of irrigation for agricultural lands and is known to be contaminated with pollutants, including pesticides and human and animal waste [31]. Previous studies have reported contamination of products, such as spinach, parsley, cabbage, and lettuce, collected from the Beqaa Valley with fecal bacteria [32]. Considering that fresh produce is often consumed raw, the risk of foodborne diseases in the Northern region is heightened.

Physicochemical characteristics including pH, temperature, conductivity, and turbidity can significantly impact the aquatic ecology. Water temperature is influenced by local climate and plays a crucial role in aquatic ecosystems, influencing various physicochemical characteristics of the water environment and affecting the reproduction and metabolism of aquatic organisms [33]. The variation in pH along different sampling points could be attributed to the geological composition of the riverbed and pollution through surface runoff. In addition, turbidity levels increased from upstream to downstream sites, exceeding the guideline values. This difference may be due to soil erosion and water runoff from human activities. The highest average DO levels were observed during the dry sample period, which could be attributed to nutrient enrichment from nearby fields and the discharge of chemicals or pollutants from surrounding industries. During the dry season, the BOD5 content is lower than in the wet season due to increased surface runoff and rainfall discharge into the water. Moreover, excessive bacterial load in water indicates high BOD5 levels, which can result from the consumption of dissolved oxygen, leading to a decrease in DO concentration values. Consequently, fish and other aquatic organisms of Lebanese rivers may struggle to survive in such water conditions [34]. The Pearson correlation coefficient (r) is a statistical tool used to assess the relationship between different water quality parameters by measuring the strength and direction of the linear relationship between variables [35]. In this study, strong positive correlations were observed during both seasons between bacterial contaminants (total coliforms, fecal coliforms, Enterococci) and water quality parameters like BOD5 and turbidity. Additionally, DO levels appear to be negatively affected by bacterial contamination. Temperature seems to influence BOD5 and turbidity positively, suggesting that temperature may similarly influence water quality parameters throughout the year. High correlation coefficients between variables indicate that they share similar sources of contamination or undergo similar geochemical processes. Furthermore, the correlation strength differences between the seasons may be attributed to environmental factors, such as rainfall and runoff or increased water quantity in the wet season, that influence water quality.

The presence of fecal contamination in water samples was assessed by isolating *E. coli* strains, as an indicator of fecal contamination and the potential presence of other pathogenic microorganisms living in communities. Moreover, the isolated antibiotic-resistant *E. coli* in surface water could be an important carrier and a rich source of emerging and spreading antimicrobial-resistant determinants to other surrounding counterparts [36]. *E. coli* strains can be classified into different phylogroups, namely, A, B1, B2, C, D, E, and F. Phylogroups A and B1 are sister groups, while phylogroups B2 and D are known to be more virulent [37]. It should be highlighted that isolates from aquatic habitats have been detected in phylogenetic groups A and B1 more frequently than isolates from phylogenetic groups B2 and D [38]. In this study, phylogroup B2 was the most prevalent, followed by phylogroups A, D, and E, respectively. *E. coli* strains related to the B2 and D phylogroups carry more virulence-associated genes compared to the other phylogroups and are primarily associated with extraintestinal infections, including urinary tract infections [37]. However, our findings are in contrast with previous studies conducted in Portugal, Malaysia, and Iraq that showed a predominance of isolates belonging to the A and B1 phylogenetic groups [39]–[41]. Moreover, a recent study conducted by Diab et al. [21] reported that phylogroup A was the most prevalent in Lebanon's water, while phylogroup B2 was the least prevalent. These discrepancies could be attributed to differences in water sources (wells, springs, estuaries, or rivers) and geographical locations.

Given the release of untreated sewage and other contaminants into Lebanese surface water and the widespread ARB in other crucial matrices in Lebanon [42], addressing ARB in our samples was necessary. Moreover, our findings showed that approximately 56/100 (56%) of the *E. coli* isolates were MDR, which is consistent with the findings of Bong et al. [36], who reported the presence of MDR ESBLs in anthropogenic-impacted rivers. The percentage of MDR *E. coli* in Lebanese surface water is slightly lower than what has been reported for sewage-contaminated rivers in Romania (60.34%) and Ethiopia (78%) [43],[44]. However, considering that Lebanon is one of the smallest countries in Asia in terms of total area (~10,450 km^2^) and population (~6.8 million), the prevalence of MDR *E. coli* in surface water is still a significant concern. Our finding aligns with a previous study by Tokajian et al. [16], which examined bacterial loads in sewage-contaminated surface water samples from Lebanon. In another study, ESBL-producing *E. coli* constituted 77.3% of all isolates and was also the predominant organism in various water sources in Lebanon, including wells, estuaries, and rivers [21]. In Lebanon, the increasing prevalence of antibiotic-resistant bacteria in surface water has become a growing concern due to the absence of efficient strategies for collection and domestic wastewater management. The Central Administration of Statistics reports that only 37% of buildings in Lebanon are connected to a sewer network, while the rest rely on cesspools and septic tanks or directly release untreated sewage into the environment [22]. The North district, which hosts a large number of refugees, also encompasses the most impoverished and vulnerable communities. Moreover, 30% of these refugees reside in densely populated areas, exacerbating issues such as overcrowding, infrastructure damage, and limited access to water and sanitation services [22]. Consequently, this population increase has intensified the pressure on various resources in Lebanon, including water. According to the International Institute for Environment and Development, there has been a 30% increase in water demand since the onset of the Syrian conflict. Additionally, research published by the Ministry of Environment in 2014 indicated a 33% rise in wastewater pollution since 2011, which can be attributed to the population growth around water sources [22].

All the ESBL-producing *E. coli* isolates in this study exhibited resistance to third-generation cephalosporins and aztreonam. Thus, we have investigated the distribution and characterization of ESBL-producing *E. coli*. Interestingly, 62% of strains showed additional resistance to cefoxitin. The resistance patterns varied within the different phylogroups. The phylogroups B2 and D exhibited lower levels of antimicrobial resistance compared to the phylogroups A and B1 [45]. These findings reflect the influence of antibiotic overuse and inadequate wastewater management, which contribute to the development and spread of antimicrobial resistance in the environment. We then investigated the presence of ESBL-encoding genes in these isolates, including bla_CTX-M_, bla_TEM_, bla_OXA_, and bla_SHV_. The bla_CTX-M_, bla_TEM_, and bla_SHV_ genes were more prevalent than bla_OXA_. These findings are consistent with a recent study that reported the recovery of ESBL-producing *E. coli* from natural water sources [46]. The presence of bla_CTX-M_ genes in water is often associated with highly self-transferable plasmids, indicating the transfer of ESBL-producing *E. coli* from sewage to rivers [47]. The most common ESBL gene identified in our study was bla_CTX-M-15_ (32/56, 57%), which is consistent with recent studies conducted in South Africa and Portugal and with studies in Lebanon covering estuaries, wells, springs, and rivers [16],[21],[48],[49]. This frequency is higher than that shown in Thailand (44.7%) [50], but a higher prevalence was reported in Tunisia (85%) and Germany (81%) [51],[52]. bla_CTX-M-15_ is also the predominant ESBL gene in Lebanon and has been detected in isolates recovered from animals as well as in healthy and hospitalized patients [21],[53]. The presence of untreated sewage and industrial water in surface water may contribute to the high prevalence of this resistance determinant [42]. The dissemination of bla_CTX-M-15_ has been globally linked to the spread of highly virulent strains of *E. coli*, such as ST131 (belonging to the B2 phylogenetic group and serotype O25:H4) and ST405 (phylogroup D) [54]. Co-occurrence of bla_CTX-M-15_ with other ESBL genes (bla_TEM_, bla_SHV_, or bla_OXA-48_) was also observed in 32/56 (57%) of the MDR isolates in our study. This phenomenon is common among Gram-negative organisms isolated from surface water [55]. In addition to bla_CTX-M-15_, bla_TEM_ was found in 36/56 (64%) of isolates. This gene has been commonly detected in sewage and river water samples in different countries such as Poland and China and even in river water samples from Lebanon [16],[56],[57]. Previous studies have shown that bla_TEM_ has been frequently associated with IncF plasmids, implicated in the dissemination of resistance determinants, and widely distributed among Enterobacteriaceae [16]. The presence of this gene indicates the contamination of surface waters by antibiotic-resistant organisms originating from human activities or hospital effluents, as they are a major source of the bla_TEM_ gene [55]. Moreover, the carbapenemase gene, bla_OXA-48_, was observed in 11/56 (20%) of the ESBL-producing *E. coli* isolates. This finding is greater than other recent studies that reported the presence of gene encoding in 3.9% of US drinking water and 4% of water bodies in India [55],[58] but less than Kuwait, where bla_OXA-48_ was found in 30% of marine environment samples [59]. In Lebanon, carbapenemase-producing *E. coli* were recovered from water samples with bla_OXA-48_ being the most commonly identified gene [21]. This gene is commonly associated with the spread of carbapenem resistance and has been reported in Enterobacteriaceae from various sources, including humans, animals, and the environment [60]. The bla_CTX-M-15_ gene was co-present with bla_TEM_, bla_SHV_, and bla_OXA_ genes, thus promoting the evolution and dissemination of MDR and extensively drug-resistant (XDR) strains [61]. It has been reported that bacteria may integrate ARGs as residential genes in their chromosome, ensuring a stable propagation of ESBL Enterobacteriaceae [62].

Moreover, we aimed to study the phylogenetic classification of ESBL-producing *E. coli* isolates and investigate their antimicrobial resistance patterns for further information on the regional *E. coli* phylogenetic grouping and association between the phylogenetic groups and their antimicrobial resistance patterns. In the present study, the highest MDR frequency was detected in phylogenetic group B2, followed by groups A and D. Lower resistance levels were observed in isolates belonging to group E. In contrast, the highest MDR frequency was found in phylogenetic group A in Portugal, Malaysia, and Iraq [39]–[41]. In addition, there was lower prevalence of MDR in isolates in phylogenetic group B2 in Spain [63]. The disparity in these findings may be explained by the geographical differences as well as the origins of isolates found at various locations.

Although this study had limitations in terms of the number of resistance genes studied, the observed patterns of genetic resistance are intriguing and motivate more comprehensive investigations. Hence, future studies with higher sample size should consider various ESBL-producing coliforms. This will provide a deeper understanding of the current distribution of resistance genes and their dissemination among different organisms. Further genomic analyses of clinical and environmental isolates are of the utmost importance to uncover possible circulation of strains between the hospital and the community settings.

## 5. Conclusions

Antibiotics are consistently used for medical and agricultural purposes. These antibiotics are excreted from humans and animals unmetabolized, leading to the emergence of ARB, which are then disseminated by water to humans and animals. Our study highlights the wide spread of antibiotic-resistant bacteria and resistance genes in surface water in Northern Lebanon, particularly in areas with inadequate wastewater management and high population density. This situation is alarming since ARB poses a potential threat to public health, and surface water is directly intended for human consumption without any further treatment or is used to water animals and irrigate crops. These findings highlight the urgency for implementing collaborative strategies between the water, health, environment, and agricultural sectors to reduce human exposure to pathogens and pinpoint the causes of environmental pollution, including the alarming issue of antimicrobial resistance. This is in line with the One Health project, spearheaded by the WHO, which includes actions to safeguard the wellbeing of humans, animals, and ecosystems. Sustainable solutions such as improved wastewater treatment systems and proper sanitation infrastructure are required. Additionally, monitoring antibiotic use in human health, but also in animal health, and implementing antibiotic stewardship programs can help reduce the selection pressure for resistance and preserve the effectiveness of antibiotics.

**Use of AI tools declaration**

The authors declare they have not used Artificial Intelligence (AI) tools in the creation of this article.
